# Coracobrachialis muscle morphology and coexisted neural variants: a cadaveric case series

**DOI:** 10.1007/s00276-023-03207-7

**Published:** 2023-07-18

**Authors:** Maria Piagkou, George Tsakotos, George Triantafyllou, Christos Koutserimpas, Dimitrios Chytas, Vasilios Karampelias, Ioannis Pantekidis, Anastasia Triantafyllou, Konstantinos Natsis

**Affiliations:** 1 Department of Anatomy, School of Medicine, Faculty of Health, and Sciences, National and Kapodistrian University of Athens, 75 Mikras Asias Str., Goudi, 11527 Athens, Greece; 2 Department of Orthopaedics and Traumatology, “251” Hellenic Air Force General Hospital of Athens, Athens, Greece; 3 Basic Sciences Laboratory, Department of Physiotherapy, University of Peloponnese, Sparta, Greece; 4 European University Cyprus, Engomi, Nicosia, Cyprus; 5 Department of Anatomy and Surgical Anatomy, School of Medicine, Faculty of Health and Sciences, Aristotle University of Thessaloniki, Thessaloniki, Greece

**Keywords:** Coracobrachialis muscle, Musculocutaneous nerve, Course, Morphology, Variation, Clinical significance

## Abstract

**Purpose:**

The current cadaveric case series evaluates the coracobrachialis muscle morphology, the related musculocutaneous nerve origin, course, and branching pattern, as well as associated adjacent neuromuscular variants.

**Materials and methods:**

Twenty-seven (24 paired and 3 unpaired) cadaveric arms were dissected to identify the coracobrachialis possible variants with emphasis on the musculocutaneous nerve course and coexisted neural variants.

**Results:**

Four morphological types of the coracobrachialis were identified: a two-headed muscle in 62.96% (17/27 arms), a three-headed in 22.2% (6/27), a one-headed in 11.1% (3/27), and a four-headed in 3.7% (1 arm). A coracobrachialis variant morphology was identified in 37.04% (10/27). A three-headed biceps brachii muscle coexisted in 23.53% (4/17). Two different courses of the musculocutaneous nerve were recorded: 1. a course between coracobrachialis superficial and deep heads (in cases of two or more heads) (100%, 24/24), and 2. a medial course in case of one-headed coracobrachialis (100%, 3/3). Three neural interconnections were found: 1. the lateral cord of the brachial plexus with the medial root of the median nerve in 18.52%, 2. the musculocutaneous with the median nerve in 7.41% and 3. the radial with the ulnar nerve in 3.71%. Duplication of the lateral root of the median nerve was identified in 11.1%.

**Conclusions:**

The knowledge of the morphology of the muscles of the anterior arm compartment, especially the coracobrachialis variant morphology and the related musculocutaneous nerve variable course, is of paramount importance for surgeons. Careful dissection and knowledge of relatively common variants play a significant role in reducing iatrogenic injury.

## Introduction

The coracobrachialis muscle (CB), located in the anterior arm compartment, originates from the apex of the coracoid process (CP) of the scapula and the proximal portion of the origin of the tendon of the biceps brachii muscle (BB). The CB is perforated and innervated by the musculocutaneous nerve (MCN) that originates from the lateral cord of the brachial plexus [29]. CB has many functions mainly at the shoulder joint. It both moves the humerus forward, causing the arm’s flexion, and toward the torso, causing the arm’s adduction. To a lesser degree, it turns the humerus inwards, causing internal rotation. In addition, CB adds to shoulder joint stabilization [2].

The CB superficial head(s) may originate from the CP, and/or the medial border of the tendon of the BB short head. CB deep head(s) may arise from the lateral border of the BB short head (CP base) [7]. Superficial and deep heads after a small course, fuse, and may entrap the MCN coursing between them. The MCN before piercing CB, may give proximal branches, the so-called accessory muscle’s innervation. In cases of an MCN non-piercing CB, it looks like the CB deep head is missing [7].

Several procedures include the muscles’ mobilization attached into the CP, mainly for treating the anterior shoulder instability [6, 12, 27], and the CB transfer in reconstructive surgery [13, 16]. The MCN may be injured also in the commonly used shoulder surgery anterior deltopectoral approach [19, 30]. Hence, MCN protection is of utmost importance in these operations [10, 18, 20], while CB typical and variant morphology, as well as associated neural variants may play a significant role.

The current cadaveric case series evaluates CB typical and variant morphology, and the related MCN origin, course, and branching pattern, as well as associated neuromuscular variants of the area. A comparison of the current findings with those of other studies in different populations is performed in an effort to identify differences.

## Materials and methods

A dissection of 15 formalin-embalmed donated cadavers of Greek origin (12 bilateral and 3 unilateral) was performed in the axilla and arm to identify CB possible morphologic variants with emphasis on the MCN course and coexisted neuromuscular variants. Skin, subcutaneous fat and the upper limb superficial fascia were dissected, and the CB was exposed from its proximal to distal attachment, to be carefully examined for its morphological variants, variant innervation, and its relationship with the MCN. The cadavers were donated to the Anatomy Department of the Medical School of the National and Kapodistrian University of Athens and the Anatomy and Surgical Anatomy Department of the Medical School of the Aristotle University of Thessaloniki, through the “Anatomical Gift Program” after written informed consent.

## Results

**Fig. 1:**
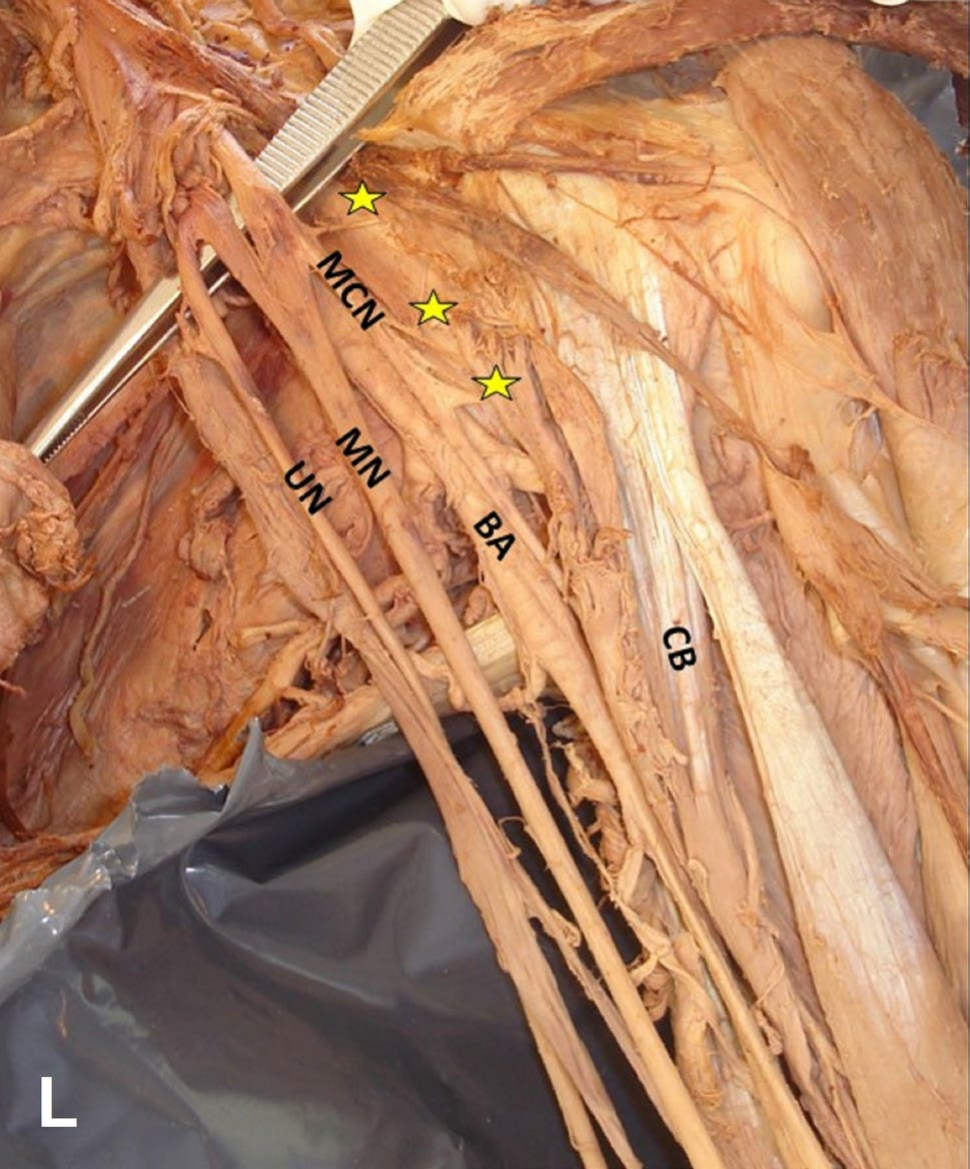
A left-sided (L) accessory innervation of the coracobrachialis muscle (CB) by the musculocutaneous nerve (MCN) proximal three branches (1, 2, 3 depicting with yellow asterisks), before the MCN piercing the muscle, *MN* median nerve, *UN* ulnar nerve, *BA* brachial artery

**Fig. 2:**
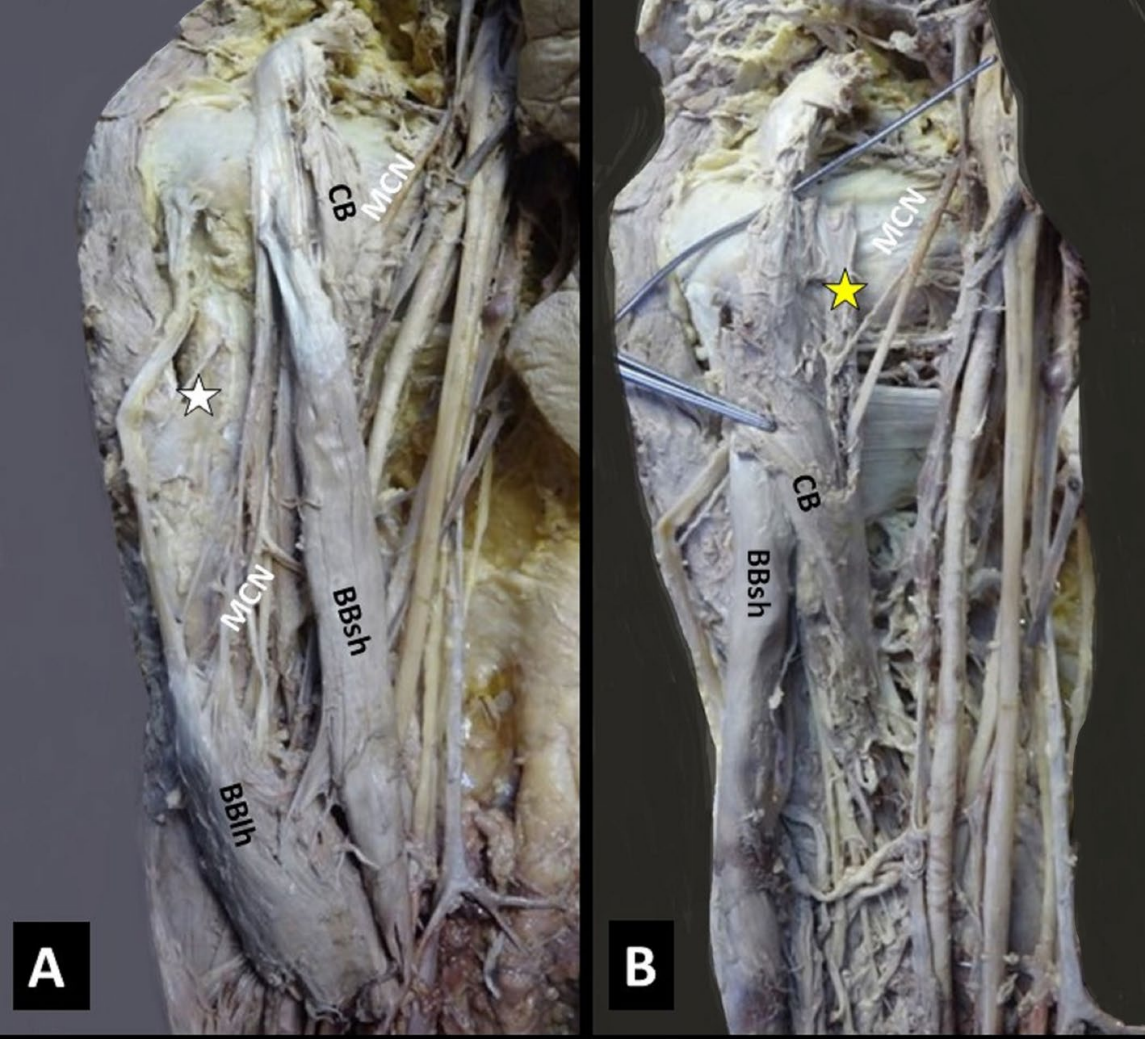
**A**, **B** In the right arm (R), the two-headed CB coexisted with two asymmetrical bellies of the biceps brachii (BB). BB short head (BBsh) was more elongated (20.2 cm length) than the long head (BBlh) (10.4 cm length). **A** Tendinous fibers of the long head originated from the humeral shaft (white asterisk), **B** MCN proximal branch (yellow asterisk)

**Fig. 3:**
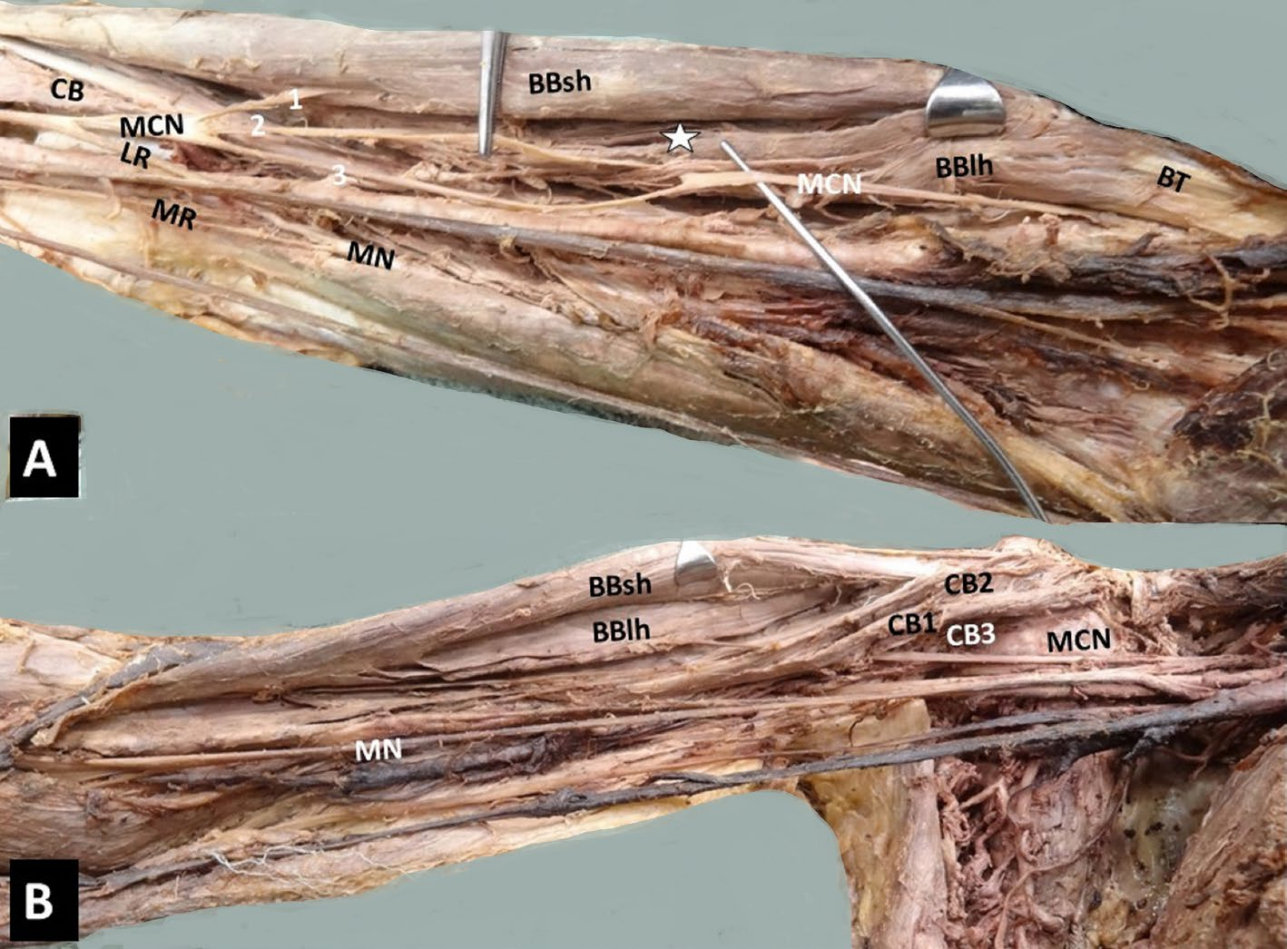
**B** Three-headed CB (right side) coexisted with (**A**). One-headed CB (left side). In the left arm, a musculocutaneous nerve (MCN) of a medial course was identified and trifurcated into branches for the innervation of the biceps brachii short head (BBsh) and long head (BBlh), while the two main trunks continued their course and after giving off branches to brachialis, they joined, creating a loop around the BB long head fibers. **A** Interconnection of the MCN with the median nerve (MN) in the middle third of humerus, muscular slip of the BB. **B** 1, 2, 3 three heads originating in order from the coracoid process, the BBsh (superficial heads) and the deep one from the CP base

**Fig. 4:**
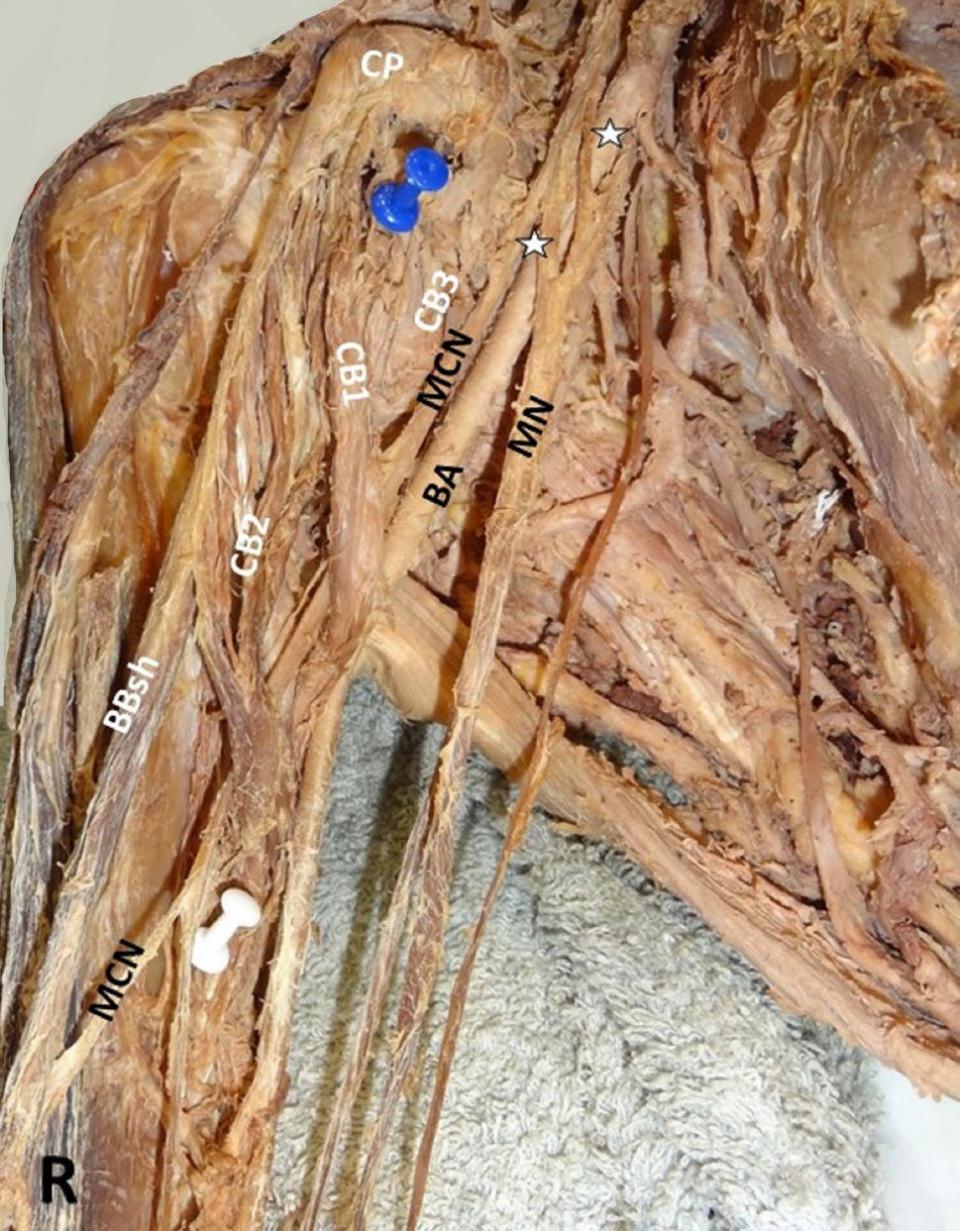
A right-sided (R) three-headed coracobrachialis muscle (CB), the 1st superficial head (CB1) emerged from the coracoid process (CP) tip and the 2nd superficial head (CB2) originated from the medial border of the tendon of the biceps brachii (BB) short head (BBsh). CB deep head (CB3) originated partially from the CP base and from the area of the lesser tubercle of humerus, MCN-musculocutaneous nerve, BA-brachial artery, MN-median nerve, asterisks the lateral root of the MN duplication. Two asterisks depicting the lateral root of the MN duplication

Four morphological types of the CB were summarized in Table 1 with their co-variants: 1. A two-headed CB (Figs. 1L, 2A, B) in 62.96% (i.e., CB typical morphology, identified in 17/27 arms, in 5 cadavers bilaterally and in 7 arms unilaterally), 2. A three-headed CB (Figs. 3B, 4R) in 22.2% (6/27, in 4 cases unilaterally and in 1 case bilaterally), 3. A one-headed CB (Fig. 3A) in 11.1% (3/27), and 4. A four-headed CB in 3.7% (1 case). A variant CB morphology was identified in 37.04% (10/27 cases). A unilateral three-headed BB coexisted in 23.53% (4/17 cases).

**Table 1:** Summary of cases’ description (age is expressed in years)

Cases C, A	Age, gender	CB morphology	Origin: CP tip or base, BBsh, BBlh	Typical insertion	MCN course Between CB heads (1) Medial to CB (2)	Coexisted variants
		Number of heads (1, 2, 3, 4)				
1C	72, M	2 (B)	Typical	In all cases	1	Unilateral three-headed BB (R) Unilateral duplication of LR (R) Bilateral interconnection LC-MR
2A	78, F	2 (U)	Typical		1	Three-headed BB Two interconnections MT-MN and UN
3C	74, M	2 (B)	Typical		1	Unilateral three-headed BB MCN-MN interconnection
4C	75, M	2 (B)	Typical		1	Unilateral MCN proximal division, and MCN-MN interconnection
5A	74, F	2 (U)	Typical		1	Unilateral duplication of LR
6A	76, M	2 (U)	Deep head/CP base and BBlh		1	BB asymmetrical bellies (sh > lh in length)
7C	74, M	2 (L) 3 (R)	Accessory head/CP tip and tendon of the BBsh		1 1	LC-MR and UN (L), LR-UN (R) interconnections
8C	72, M	1 (L) 3 (R)	Accessory head/CP tip and medial border of BBsh		2 (L) 1 (R)	Loop between main MCN and MCN branch with BBlh fibers passing through (L)
9C	80, F	2 (B)	Typical		1 (R, L)	Myofascial tunnel of BBsh and brachial fascia (L) LT-MR (R), LC-MR (L) interconnections
10C	75, F	2 (L) 3 (R)	Accessory head/tendon of BBsh		1 (R, L)	Duplication of LR (R)
11C	74, M	2 (B)	Typical		1 (R, L)	Unilateral interconnection LC-MR, contralateral UN-RN, and LC-MC interconnections
12C	80, M	3 (R) 4 (L)	Typical		1 (R, L)	Myofascial fibers between BBsh and brachial fascia (R), LC pierced CB (R)
13C	73, M	2 (L) 1 (R)	Typical		2 (L) 1 (R)	Unilateral three-headed BB
14C	76, M	1 (L) 2 (R)	Typical		1 (L) 2 (R)	None
15C	74, F	3 (B)	Accessory heads/ CP tip and tendon of the BBsh		1 (R, L)	MCN-MN interconnection (R)

Coracobrachialis muscle (CB) typical origin considered coracoid process (CP) tip and the tendon of the short head of the biceps brachii muscle (BB), also typical insertion considered the middle surface of humerus [7, 14]

*L* left, *R* right, *B* bilateral, *U* unilateral, *M* male, *F* female, *MCN* musculocutaneous nerve, *MN* median nerve, *UN* ulnar nerve, *BBsh* biceps brachii short head, *BBlh* biceps brachii long head, *LT of BP* lateral trunk of brachial plexus, *LC of BP* lateral cord of brachial plexus, *MR of MN* medial root of the median nerve, *LR of MN* lateral root of the median nerve, *MT* middle trunk nerve, *C* cadaver, *A* arm

### Musculocutaneous nerve (MCN) variant course

Two different courses of the MCN were recorded: 1. a course between CB superficial and deep heads (in cases of two or more CB heads) (100%, 24/24), and 2. a medial course in a one-headed CB (100%, 3/3).

### Muscles’ fusion via bundles

Muscle and myofascial bundles were identified in 2 cases: 1. between CB and the triceps brachii medial head, and 2. between the BB short head and the upper arm fascia.

### Neural interconnections

**Fig. 5:**
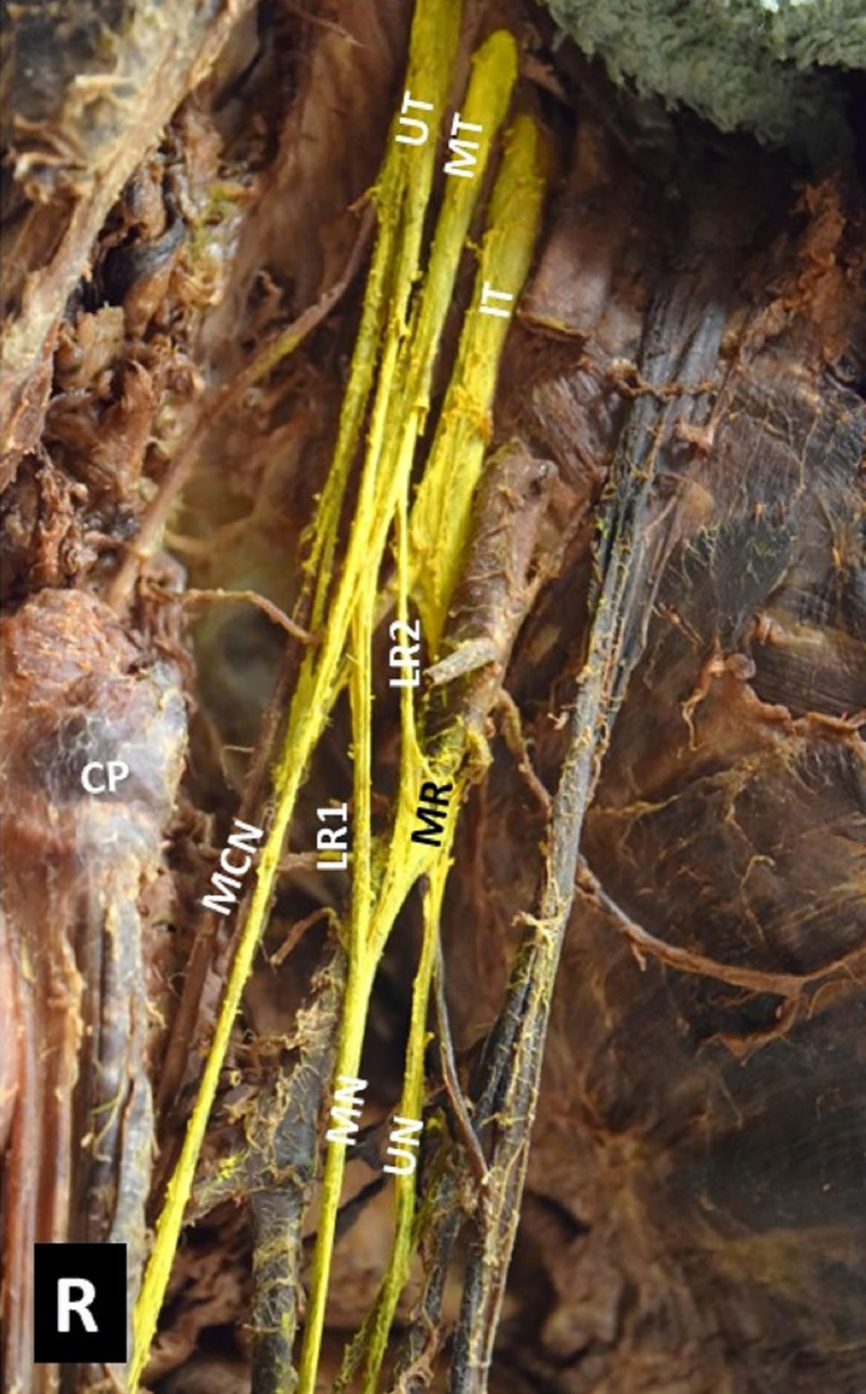
A right-sided (R) interconnection of the lateral cord (LC) of the brachial plexus (the middle trunk contribution-MT) with the medial root (MR) of the median nerve (MN) and the ulnar nerve (UN) (LR2- second lateral root), at the emersion of the thoracoacromial and lateral thoracic arteries. A MN alternatively characterized as a MN with double lateral roots (LR1 and LR2)

Three neural interconnections (ICs) were identified: 1. IC of the lateral cord of the brachial plexus with the medial root of the median nerve (MN) (Fig. 5) in 18.52% (5/27 cases, 3 unilaterally and 1 bilaterally), 2. IC of the MCN with the MN in 7.41% (2/27 cases), and 3. IC of the radial nerve with the ulnar nerve in 3.71% (1/27).

### Other neural co-variants

**Fig. 6:**
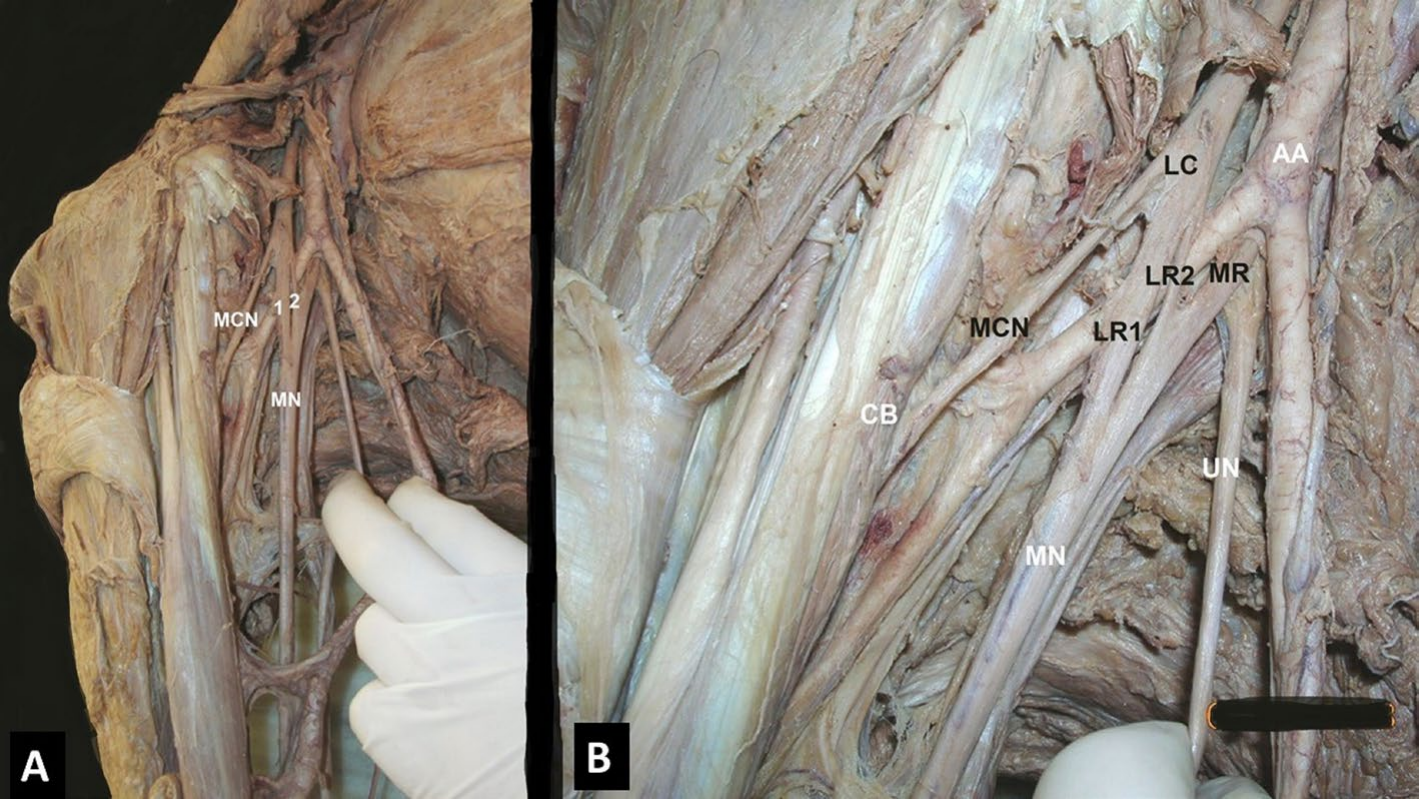
**A**, **B** The duplication of the lateral root (LR) of the median nerve (MN) (1, 2) **B** LR1, LR2 coexisted with an axillary (AA) bifurcation into a superficial and a deep stem, *LC* lateral cord, *MR* medial root, *UN* ulnar nerve, *CB* coracobrachialis muscle

A duplication of the MN lateral root was identified in 11.1% (3/27) (Fig. 6A, B).

## Discussion

The current case series identified the typical and variant CB morphology, by recording four types: type 1-a CB with two heads (one superficial and deep head, typical morphology), type 2-a CB with three heads (two superficial and one deep head), type 3-a CB with a single head, and type 4-a CB with four heads (3 superficial and one deep). Symmetrical CBs (two or three heads) were identified in 50% (6/12) and asymmetrical CBs in 50% (6/12). In the current series, the most common type was the two-headed CB in 62.96%, similar to El-Naggar [7] who recorded in almost all cases a two-headed CB. El-Naggar [7] and El Naggar and Zahir [8] were the first to suggest the CB typical two-headed nature, contrariwise to Szewczyk et al. [26] who identified a CB single belly as the most common type (49.5%), a double-headed CB in 42.6% and a three-headed CB in 7.9%. A remarkable difference concerning the most identified morphological CB variant can be pointed out between the current study (the two-headed CB, the commonest variant) and that by Szewczyk et al. (the one-headed CB, the commonest variant) [26]. The differences were explained by the different examined populations, in Szewczyk et al. [26] study, the cadavers belonged to a Central European population, while the cadavers of the current study had a Greek origin. Different populations may be correlated with differences in the topographical anatomy, as it can be confirmed by the study by Ilayperuma et al. [14] who included cadavers of Sri Lankan individuals and by the study by Larrotta et al. [15], who comprised cadavers of Colombian origin. The current series recorded two cases of muscle and myofascial bundles joining: 1. CB and triceps brachii medial head, and 2. the BB short head and the upper arm fascia. These formations narrowed the available surface area and possibly entrapped the adjacent nerves (MCN, and MN) and the brachial artery. In addition, the current series highlights the coexistence of the CB variant morphology with the BB morphologic variants in 14.8% similar to other reports [5, 8, 21, 23]. Although, CB and BB variants seem to coexist, no systematic study until now has identified this association that possibly is explained by the muscles’ developmental background. During the 4th–7th week of the embryonic development, mesenchymal cells migrate from the dorsolateral part of somites to the limb bud to create muscles [3]. The anterior arm muscles (CB, BB, and brachialis muscle) probably derive from a single mass in the embryo of 11-mm long and they can be recognized in the embryo of 14–16 mm in length, while the formation of additional heads takes place at the separation stage in an embryonal length 11–19 mm [1]. The muscles’ formation is completed before nerves’ formation. Thus, “the nerve follows the muscle”, and as a result, a developmental problem in muscle differentiation leads to the atypical innervation [4, 15, 28]. After the formation of the muscles and nerves supplying the entire limb, a plexus begins to create in the form of loops between nerve fibers, and in this way the MCN originates from the MN [3], late during arm development. Disruption in the brachial plexus differentiation may lead to the MCN absence [4, 15, 28].

In the current case series, an MCN perforating CB was observed in 88.9%, while a medial course was identified in 11.1%. Guerri-Guttenberg and Ingolotti [11], recorded a MCN not piercing CB in 11.1%, closest to the current results, while in Remerand et al. [24] ultrasound study on 388 axillae, a MCN of medial course was identified in 22%. In addition, Ilayperuma et al. [14] identified the MCN perforating CB in 83.38% and a no perforation in 16.67%. The MCN perforation easily distinguished CB superficial from deep heads. However, the relationship between the MCN course and the number of CB heads has not been systematically studied. In the current series, in 100% of the double-headed (or more) CB, the MCN coursed between the heads and in 100% of one-headed CB, the MCN coursed medially to the muscle.

In the current case series, CB variants coexisted with neural aberrations in 59.26% (16/27 arms). The IC of the MCN with the MN was identified in 11.1% (3/27 cases) of the present series, while this variant has been reported in up to 43% of cases by Guerri-Guttenberg and Ingolotti [11]. Other neural co-variants, found in the current case series, were the IC between the lateral cord of the brachial plexus and the medial root of the MN (4/27; 14.8%) and the IC of the lateral cord of the brachial plexus with the UN (2/27; 7.4%). Those variations’ prevalence has not been studied in the literature. In addition, the lateral root duplication was observed in 11.1% (3/27, all cases unilaterally) by the current case series, while Pandey and Shukla [22] recorded a lower prevalence of 4.65% (8/172 cadavers). Some studies recorded the presence of accessory branches originating at a high-level position from the MCN main trunk [10, 17, 25]. Although this research point needs further investigation from larger clinical studies, surgeons need to know this correlation when operating, as the MCN lesions result in reduced elbow flexion strength and sensory impairment of the forearm radial aspect.

Variants of the anterior arm compartment, such as the CB accessory heads or the aberrant MCN course, have clinical complications due to high number of surgical procedures performed in the area. The Latarjet procedure is frequently used to treat recurrent anterior shoulder instability with more than 20% bone loss of the humeral glenoid. Latarjet, before using this technique, studied the MCN anatomy in relation to CP and CB [20] and identified the MCN proximal branches, before the CB piercing by the MCN, and identified the cause of neurovascular injury after the procedure. El-Naggar’s study [7], several years later, claimed the separate innervation for the two-headed CB (isolated for each head), so the presence of accessory branches is clinically important. Also, orthopedic surgeons should take into consideration the bilateral existence of CB variants in 50% in the current study. It is of note that during the deltopectoral approach which is utilized for most of the open shoulder surgery, the surgeon should not extend the approach medially of the CP. There are also other procedures involving the area of the CP, such as acromioclavicular dislocations and fixations of CP fracture. Careful surgical technique and dissection of the area are of utmost importance adequate surgical visualization and injury avoidance. Moreover, in complex cases, preoperative imaging may be considered to avoid iatrogenic intraoperative lesions [9].

The presence of variant muscles of the anterior arm compartment may cause neuropathy, such as MCN neuropathy due to muscular compression. The most common site of MCN entrapment is the CB and the presence of additional heads [21]. In 88.9% of arms in the current series, the MCN coursed through the muscle, so there was a great chance of compression leading to numbness or deficit on the glenohumeral joint [21]. Also in two cases, muscle and myofascial fibers were identified, passing anterior to the brachial artery and the MN, thus probably compressing on them, and resulting in symptoms.

## Limitations

As the cadaveric case series were used for the students’ educational purposes, we could not perform a detailed morphological, as well as a morphometric study. Thus, we didn’t obtain data about the vascular variants of the area. The cadavers were initially dissected by students and further by the senior authors, that’s why this case series does not conclude the co-variants regarding the area vascularization, as well as data concerning the MCN proximal branching pattern before the entrance to CB. In addition, although the gender and side of investigation were known, their impact on muscle’s morphology was not recorded due to the low number of dissected parts. Finally, the subjects’ medical history was unknown.

## Conclusions

Muscle variants of the anterior arm compartment are not rare, as well as coexisted muscles and neurovascular aberrations. A relation exists between the CB typical and variant morphology and the MCN course. The knowledge of accessory CB heads and/or muscle bundles joining the muscles of the anterior arm compartment is clinically important because they may compress on the MCN, and/or the MN during their course between them, leading mainly to numbness. Also, cases of atypical MCN course, medial to CB, may complicate imaging leading to false interpretation. Alterations of the brachial plexus arrangement and occurrence of ICs between the nerves, or branches duplication are variants, essential to know in order to avoid iatrogenic injury, intraoperatively.

## Data Availability

Data and material related to the report will be available with the corresponding author for further reference.

## Abbreviations

CB: Coracobrachialis muscle
BB: Biceps brachii muscle
CP: Coracoid process
MCN: Musculocutaneous nerve
MN: Median nerve
IC: Interconnection
